# Development and validation of a modified Korean version of the Pharmacy Services Questionnaire (PSQ-K) for the quality assessment of community pharmacy services

**DOI:** 10.1371/journal.pone.0174004

**Published:** 2017-04-25

**Authors:** Kyung Im Kim, Hae Sun Suh, Arim Kwak, Siin Kim, Nayoung Han, Euni Lee, Jung Mi Oh

**Affiliations:** 1 College of Pharmacy, Korea University, Sejong, Republic of Korea; 2 Biomedical Research Center, Korea University Guro Hospital, Seoul, Republic of Korea; 3 College of Pharmacy, Pusan National University, Busan, Republic of Korea; 4 Research Institute of Pharmaceutical Sciences, Seoul National University, Seoul, Republic of Korea; 5 College of Pharmacy, Seoul National University, Seoul, Republic of Korea; University of Glasgow, UNITED KINGDOM

## Abstract

Patient-reported outcome (PRO) measures and validated instruments have become integral in assessing the quality of healthcare delivery, including pharmaceutical care services. The Pharmacy Services Questionnaire (PSQ) measures patient satisfaction with pharmaceutical care. In this study, we developed a modified Korean version of the PSQ (PSQ-K) and evaluated its validity and reliability. The PSQ-K was developed using a strict translation and cultural-adaptation procedure. A validation study was performed in six community pharmacies in Korea. A total of 300 respondents completed three questionnaires (a brief questionnaire for social demographics and clinical characteristics, the PSQ-K, and the 5-level EuroQoL Group’s 5-dimension [EQ-5D-5L]). Standard validity and reliability analyses were performed. The internal consistency of the PSQ-K was high for all scales (Cronbach’s α > 0.9). The PSQ-K indicated good discriminant and divergent validity. Known-group comparisons revealed that the PSQ-K was able to distinguish between respondents differing in socio-demographic characteristics, such as gender, level of education, and household income. In conclusion, the PSQ-K is a highly reliable and valid PRO instrument for assessing the level of satisfaction with community pharmacy services.

## Introduction

Reports that come directly from patients regarding the status of their health and quality of life, i.e., patient-reported outcomes (PRO), have been considered as a critical outcome for the assessment of the quality of healthcare [1, 2]. In addition, patient satisfaction can serve as an ultimate outcome indicating quality of healthcare services and has been an essential part of quality assessment [1, 3]. A standardized PRO instrument for patient satisfaction and healthcare providers has addressed patients’ satisfaction regarding pain management and communication about medicines, which are important services provided by pharmacists [4].

There are a few PRO instruments estimating patients’ satisfaction with pharmaceutical care services, e.g., Pharmacy Services Questionnaire (PSQ) [5], Pharmacist Services Questionnaire (PSPSQ) [6], and Pharmaceutical Care Satisfaction Questionnaire (PCSQ) [7] that have demonstrated appropriate psychometric properties in English. Among them, the PSQ is the most widely used instrument, comprising 20 items in two multi-item scales (11 Friendly Explanation [FE] items and 9 Managing Therapy [MT] items) [5]. It provides a quantitative measure of patients’ evaluations of a pharmacist’s professional performance, promptness of service, relationship with the pharmacist, and how well the pharmacist explained what a medication does and how to take it.

It is important to develop and validate a questionnaire with a specific language that reflects cultural differences and the underlying meanings of phrases with respect to the original questionnaire [8]. As far, the PSQ has been translated and validated for Spanish, Portuguese, and Arabic, but not in Korean. Thus, in this study, we developed a modified Korean version of the PSQ (PSQ-K) and evaluated its psychometric properties when applied to Koreans who visit community pharmacies. To the best of our knowledge, this is the first psychometric validation study of the PSQ in Asia.

## Methods

### Development of the PSQ-K

#### Translation

**Fig 1:**
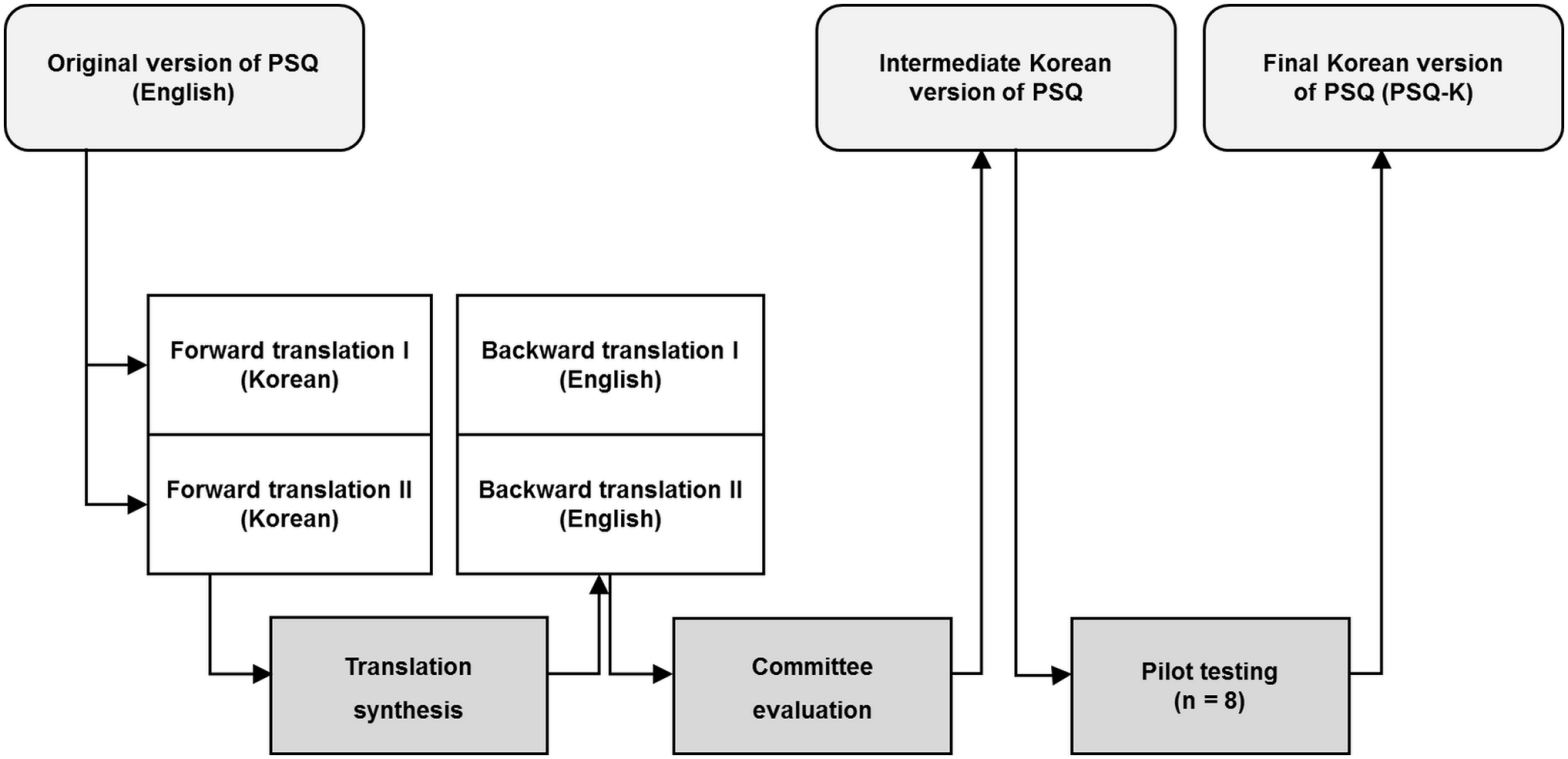
Cultural adaptation process of the Korean patient satisfaction questionnaire.

The overall translation process followed guidelines for cross-cultural adaptation (Fig 1) [9]. First, two translators (two professors of college of pharmacy, both licensed pharmacists in Korean and U.S.), fluent in both English and Korean, performed a forward translation from English into Korean independently. An investigator (KIK) convened the first consensus meeting with the translators to merge the translated’ versions into one single reconciled forward translation by choosing the most appropriate translation, combining them, or suggesting alternative translation when necessary. Second, back-translation of the reconciled version into English was done independently by another two translators who are bilingual in English and Korean (two professors of college of pharmacy), without knowledge of the original questionnaire. An investigator (KIK) and the two backward translators had the second consensus meeting to ensure content and semantic equivalence between the original and back-translated version and then made the intermediate Korean version of the PSQ. During the translation process, two items from the original PSQ (items on the promptness of prescription drug services and the professionalism of the pharmacy staff) were excluded in consideration of the differences in regulations for community pharmacy services between the United States and Korea.

#### Pilot study

The intermediate Korean version of the PSQ was pilot tested with four native Korean patients with diabetes and four lay persons who were native Koreans. The respondents were asked to fill out the intermediate Korean version of the PSQ and then they participated in face-to-face interviews to examine the respondents’ understanding of each item. A probing technique was used to find out more information if needed. The interviewer asked how the respondent understood each item, and depending on the answer, the interviewer probed for more information by asking the reasons. After gathering all information from the respondent, the interviewer went through a quick appraisal of responses. If there were similar understandings for different items, the interviewer asked “Do these two items sound similar or exactly the same?” and the reasons of the responses. After all, the interviewer asked respondents whether the items made them feel uncomfortable and why.

The analyzed results of lists of issues identified in the pilot study were discussed by the translators and an independent project manager. They made suggestions for alternative wording adapted to Korean culture to enhance the understanding of items in the questionnaire and reached the consensus to make the final modified Korean version of the PSQ.

### Validation of the PSQ-K

#### Study population

The validation process was performed with native Korean, aged 19 or older, who visited six community pharmacies in Seoul, Korea from October 2015 to January 2016. A convenience sample of the community pharmacies was used to recruit the respondents while considering geographical proximity to the top five tertiary hospitals where the national wide patients visit. Participants were recruited in the respective pharmacies waiting area while they were waiting to get their prescription filled. Study subjects who were unable to complete the questionnaire due to serious physical or psychological morbidities or those who had difficulties in communicating with investigators in Korean were excluded. Based on a recommendation of a 5:1 subject-to-item rule, a minimum of 90 respondents were required for the validation study of the PSQ-K [10]. A dropout rate of 10% would result in 100 respondents for each north, south, and east area where the big tertiary hospitals are located in Seoul.

This study was approved by the institutional review board of Seoul National University (IRB No. 1510/002-003) and was conducted in accordance with the precepts established by the Declaration of Helsinki. Signed informed consent was obtained from each respondent before study entry.

#### Data collection and instruments

The respondents were asked to complete three questionnaires (a brief questionnaire for social demographics and clinical characteristics, the PSQ-K, and the 5-level EuroQoL Group’s 5-dimension [EQ-5D-5L]). For those who experienced some difficulty in reading the questions themselves, the trained assistant helped them in filling in the questionnaires. After completion, the researcher ensured that all questions had been answered.

The PSQ-K comprises 18 items (nine items in each of the FE and MT scales), with responses ranging from excellent (5) to poor (1). Since PSQ-K measures the level of satisfaction with pharmaceutical care [5], the validated Korean version of the EQ-5D-5L was used to measure and evaluate general health status. The Korean version of the EQ-5D-5L consists of a visual analogue scale along five dimensions (mobility, self-care, usual activities, pain or discomfort, and anxiety or depression) with five levels [11].

#### Statistical analysis

For the statistical analysis, mean score of the PSQ-K and the EQ-5D-5L index were calculated following each questionnaire’s standard protocol [5, 12]. The scale structure of the PSQ-K was examined using a multitrait scaling analysis [13]. The internal consistency reliability of the PSQ-K was assessed with Cronbach’s alpha coefficients (α) as excellent if α ≥ 0.90; good if 0.70 ≤ α < 0.90; acceptable if 0.60 ≤ α < 0.70; poor if 0.50 ≤ α < 0.60; and inacceptable if α < 0.50 [14–16]. The item-convergent validity was defined as a correlation ≥ 0.40 between an item and its own scale (corrected for overlap). The item-discriminant validity was supported when the correlation between an item and its own scale (corrected for overlap) was significantly higher than its correlation with any other scale. Divergent validity was assessed by evaluating the correlations between the PSQ-K and the EQ-5D-5L scores. Pearson’s correlation coefficients were used to evaluate the associations. Known group validities were examined by comparing the scores of the PSQ-K between respondent groups differing in social demographics or clinical status. Group differences were assessed using Student’s t-tests or Mann-Whitney U-tests.

All of the statistical tests were two-sided, with the significance of the results defined as *P* < 0.05. The analysis was performed using IBM SPSS Statistics version 22.0 (IBM, Armonk, NY, USA).

## Results

### Translation and pilot study

The intermediate Korean version of the PSQ included two scales FE and MT consisted of the nine items, respectively. The FE scale consisted of the items on appearance of the pharmacy; professional relationship with the pharmacist; and how well the pharmacist answered questions, explained what a medication does and how to take it, etc. The MT scale covered the pharmacist's interest in the patient's health; the pharmacist's managing and assuming responsibility for drug therapy; and the pharmacist's efforts to ensure that medications work as intended, solve medication-related problems, and improve the patient's health.

For the pilot study, the average time spent on filling-out the intermediate Korean version of the PSQ by respondents was 4 minutes. On average, ten minutes were spent on the interview without any statistical differences by gender or age. Overall, respondents commented that the intermediate Korean version of the PSQ was easy to complete and the items were relevant. Reliability coefficients indicated highly acceptable internal consistency (α = 0.925).

### Patient demographics and clinical characteristics

A total of 300 respondents from the six community pharmacies were enrolled in this study. Table 1 summarizes the characteristics of the respondents. The mean age was 50.6 ± 16.9 years (range, 19–86), and 112 (37.3%) of them were male. More than half of the respondents (51.0%) had chronic diseases such as hypertension, dyslipidemia, diabetes, etc. Among the respondents who use chronic medications, 85 (57.0%) of respondents took their medicine once a day. Each patient required about 20 minutes to complete all three questionnaires.

**Table 1:** Socio-demographic and clinical characteristics of the respondents (N = 300).

Variable	Mean (SD, range) or N (%)
**Age, years**	50.6 (16.9, 19–86)
**Gender**	
Male	112 (37.3)
Female	188 (62.7)
**Education**	
Elementary school	21 (7.0)
Middle school	16 (5.3)
High school	56 (18.7)
College	26 (8.7)
University or above	180 (60.0)
Unknown	1 (0.3)
**Marital status**	
Married	210 (70.0)
Divorced/widowed	12 (4.0)
Single	78 (26.0)
**Household income (Korean 10,000 won/month)**	
< 400	130 (43.3)
≥ 400	123 (41.0)
Unknown	47 (15.7)
**Chronic disease**	
Yes	153 (51.0)
No	147 (49.0)
**Chronic medication use**	
Yes	149 (49.7)
No	151 (50.3)
**Number of doses/day**	
None	151 (50.3)
1	85 (28.4)
2	40 (13.3)
3	21 (7.0)
≥ 4	3 (1.0)
**Experience of pharmacist counseling in the last 3 months**	
Yes	192 (64.0)
No	108 (36.0)

### Reliability and structures

Table 2 provides the mean and standard deviation for each item in FE and MT scale. The mean FE scale score was higher than the MT scale score, indicating a higher level of satisfaction with the FE items (3.39 ± 0.79 of FE scale vs. 2.87 ± 0.90 of MT scale). The maximum possible scores (ceiling) for the FE and MT scale were observed for 2.7% (n = 8) and 1.7% (n = 5) of respondents, respectively. The lowest possible scores (floor) was never observed in any scales. The internal consistency of the PSQ-K was excellent, with α values of 0.91 and 0.93 for the FE and MT scales, respectively.

**Table 2:** Descriptive statistics and construct validity of the PSQ-K.

	Mean (SD)	Item-own scale correlation ^a^^,^ ^b^	Item-other scale correlation ^b^
**FE scale**			
Q1. The professional appearance of the service environment	3.46 (0.89)	0.68	0.54
Q2. The availability of the pharmacist to answer your questions	3.63 (1.00)	0.66	0.50
Q3. The pharmacist's professional relationship with you	3.03 (1.12)	0.65	0.60
Q4. The pharmacist's ability to advise you about problems that you might have with your medications	3.10 (1.12)	0.67	0.65
Q5. How well the pharmacist explains what your medications do	3.25 (1.06)	0.66	0.71
Q10. How well the pharmacist instructs you about how to take your medications	3.56 (1.05)	0.68	0.56
Q11. Your pharmacy services overall	3.28 (1.03)	0.77	0.75
Q12. How well the pharmacist answers your questions	3.58 (0.99)	0.74	0.66
Q14. The courtesy and respect shown you by the pharmacist	3.61 (1.04)	0.74	0.66
**MT scale**			
Q6. The pharmacist's interest in your health	2.63 (1.07)	0.78	0.68
Q7. How well the pharmacist helps you to manage your medications	2.92 (1.14)	0.73	0.65
Q8. The pharmacist's efforts to solve problems that you have with your medications	2.73 (1.09)	0.81	0.68
Q9. The responsibility that the pharmacist assumes for your drug therapy	2.83 (1.14)	0.76	0.70
Q13. The pharmacist's efforts to help you improve your health or stay healthy	2.91 (1.13)	0.83	0.71
Q15. The privacy of your conversations with the pharmacist	3.32 (1.01)	0.59	0.57
Q16. The pharmacist's efforts to assure that your medications do what they are supposed to	2.59 (1.20)	0.79	0.63
Q17. How well the pharmacist explains possible side effects	2.99 (1.17)	0.71	0.62
Q18. The amount of time that the pharmacist offers to spend with you	2.90 (1.04)	0.72	0.71

The scores of each scale range from 0 to 5, with a higher score representing a higher level of satisfaction.

^**a**^ Corrected for overlap.

^**b**^ Range of Pearson’s correlation coefficient values.

Abbreviations: FE = friendly explanation; MT = managing therapy; SD = standard deviation

### Validity

Each item of the PSQ-K correlated with its own scale with *r* ≥ 0.59, corrected for overlap, and met the recommended psychometric standards (Table 2). For discriminant validity, all items in the MT scale and almost items in the FE scale were correlated more highly with their own scale (range, 0.59–0.83) than with the other scale (range, 0.50–0.75). Only the item on pharmacist’s explanations about the medications (item 5) correlated higher with the MT scale (*r* = 0.71) than with the hypothesized FE scale (*r* = 0.66).

In analyses performed with gender as the grouping variable, there were significant differences in the scores for both scales (Table 3). There were also significant differences in the PSQ-K scores according to the respondents’ education level. The respondents with lower education levels (less than college) exhibited significantly higher satisfaction scores on the FE (*P* < 0.001) and MT scales (*P* = 0.021) than did respondents with higher education levels. Similar results were observed in the analysis performed with the level of household income (*P* = 0.033 and 0.057 for the FE and MT scales, respectively). The respondents with chronic disease or chronic medication use exhibited significantly higher scores on the FE scale, but not on the MT scale. When assessing the relationship with the EQ-5D-5L, the FE and MT scale scores of the PSQ-K demonstrated weak correlations with the EQ-5D-5L scores (*r* = 0.068 and 0.018, respectively) (Table 4).

**Table 3:** Known-group comparisons according to socio-demographic and clinical characteristics.

Variable	PSQ-K total	FE	MT
**Gender**			
Male	3.29 (0.85)*	3.51 (0.84)*	3.07 (0.94)*
Female	3.03 (0.76)*	3.32 (0.76)*	2.75 (0.85)*
**Level of education**			
Below college	3.32 (0.81)*	3.63 (0.77)**	3.02 (0.94)
University or above	3.00 (0.78)*	3.23 (0.77)**	2.77 (0.85)
**Household income (Korean 10,000 won/month)**			
< 400	3.26 (0.87)*	3.51 (0.85)*	3.00 (0.96)
≥ 400	3.04 (0.72)*	3.30 (0.70)*	2.78 (0.82)
**Chronic disease**			
Yes	3.23 (0.82)*	3.54 (0.80)*	2.93 (0.92)
No	3.02 (0.78)*	3.24 (0.75)*	2.81 (0.87)
**Chronic medication**			
Yes	3.22 (0.84)	3.52 (0.83)*	2.91 (0.94)
No	3.04 (0.76)	3.26 (0.74)*	2.83 (0.85)
**Experience of pharmacist counseling in the last 3 months**			
Yes	3.15 (0.81)	3.44 (0.80)	2.85 (0.90)
No	3.10 (0.79)	3.29 (0.77)	2.91 (0.88)

Data are mean (SD).

Abbreviations: FE = friendly explanation; MT = managing therapy

* P < 0.05,

** P < 0.001

**Table 4:** Correlations between the PSQ-K and EQ-5D-5L.

	EQ-5D-5L
**PSQ-K Total**	0.024
**Friendly explanation**	0.068
**Managing therapy**	0.018

Data are the absolute values of the Pearson’s correlation coefficient.

## Discussion

Pharmaceutical care is a patient-centered professional practice that involves the responsible provision of pharmacotherapy for the purpose of achieving definite outcomes related to the improvement of patients’ health and quality of life [17, 18]. Therefore, PRO measurements and validated instruments have become integral parts in assessing quality of pharmaceutical care services.

In this study, both the FE and MT scales of the PSQ-K showed excellent internal consistency and the mean score of the FE scale was higher than that of the MT scale (Table 1). This finding is in accordance with the results of a previous study [5]. Larson et al. conducted a study with eight community pharmacies whose pharmacists had received training in pharmaceutical care, but who had yet to implement the training. The internal consistencies of the original version were as follows: FE (α = 0.957) and MT (α = 0.962). They found that the MT items were scored lower than the FE items for all pharmacies [5]. Similarly, community pharmacy services in Korea are limited in traditional services, such as basic patient counselling or patient-friendly services. Given that most Koreans still regard the management of therapy as the role of a physician or hospital, we suggest that lower scores on the MT scale than on the FE scale may have been from a lack of awareness of the pharmacists’ role in managing therapy.

The results of scale structure analysis revealed that there were satisfactory item-scale correlations (corrected for overlap) for all items. For discriminant validity, only the item regarding the pharmacist’s explanation about medications correlated more highly with the MT scale than with its own FE scale. Regarding this result, we could speculate that the item may overlap in meaning with the MT scale in that respondents may not be able to discern between the concepts of medication counseling and MT. Larson et al. also reported a corroborating finding of a high correlation of 0.873 between the FE and MT scales [5]. Thus, the suboptimal correlations for the item-discriminant validity of the item may not significantly compromise the overall construct validity of the PSQ-K.

Known group comparison analyses showed significantly higher levels of satisfaction for male respondents. This result of our study is in line with previous studies investigating the influence of patient gender on communication in the medical visit [19, 20]. For this, the authors explained that female patients generally tended to have greater expectations from their healthcare services than did male patients and were, therefore, more likely to experience unmet needs during their visit [21, 22]. Regarding the relationship between satisfaction and level of education, some previous studies also showed that patients with less education tended to be more satisfied with healthcare services [23, 24]. In addition, we also found that respondents with chronic disease or chronic medication use exhibited significantly higher scores on the FE scale, but not on the MT scale. This discrepancy can be partially explained by the fact that the respondents with chronic disease or chronic medication use may be more familiar with the community pharmacy or their pharmacy services since they visit the community pharmacy regularly for their prescription drugs. Given that almost all community pharmacies in Korea do not yet actively provide pharmaceutical care services, one would also expect there would be no significant difference in satisfaction scores on the MT scale between the groups. As expected, the scores of the PSQ-K scales were uncorrelated with the EQ-5D-5L scores. This suggests that the satisfaction issues in pharmaceutical care addressed by the PSQ-K are distinct from the health status assessed by the EQ-5D-5L.

In the PSQ-K, a few modifications were made to the original PSQ during translation and pilot study. First, the items on the promptness of prescription drug services and the professionalism of the pharmacy staff of the original PSQ were excluded since, in contrast to the United States, pharmacy staff’s professional roles such as assisting the dispensing medicines and some prescription drug services of community pharmacies such as prescription refills are illegal in Korea. Second, there were difficulties in understanding “the professional appearance of the pharmacy” (item 1) and “the pharmacist’s professional relationship with you (item 3). These difficulties could possibly be explained by that most respondents are not familiar with the concept of professional pharmacy services or professionalism of pharmacists in Korea. Thus the wording of “pharmacy” (item 1) substituted to a different Korean wording “the service environment” which was suggested by respondents. These modifications in the PSQ-K were confirmed to reflect the Korean culture and have semantic equivalence with the original PSQ by the researchers and translators.

While our study has the strength of including a sufficient number of respondents, there are some limitations. The majority of respondents in our study appeared to have high level of literacy, as indicated by the high proportion of respondents with relatively high education level. And an examination of the responsiveness of the PSQ-K was not done because this would require a longitudinal study. Further, longitudinal studies in a variety of education levels are advised to confirm our results and assess the sensitivity and responsiveness of the PSQ-K.

In conclusion, with a PSQ-K, our study has contributed to efforts documenting patient satisfaction with community pharmacy services in Korea, using the translated and validated version of the survey, thus facilitating quality assessment of pharmacy services in the global community.
